# Healthcare inequity and digital health–A bridge for the divide, or further erosion of the chasm?

**DOI:** 10.1371/journal.pdig.0000268

**Published:** 2023-06-02

**Authors:** Yacine Hadjiat

**Affiliations:** 1 French National Institute of Health and Medical Research (INSERM) U987 Department of physiopathology and clinical pharmacology of pain, Boulogne-Billancourt, France; 2 Paris-Saclay University (EDSP) Paris, France; New York University, UNITED STATES

Healthcare technology innovations may have received a proliferation of attention during the Covid-19 pandemic, but this belies the fact that these developments have been a significant driver of change in the health sector for some time [1]. An interpretation of recent experiences has clearly indicated that it is imperative for the healthcare sector, policy makers and community workers to consider the benefits and costs of digital developments–they may promise a solution for inclusive health services but instead entrench inequality further. Equally, the opposite can be true where digital health developments promote access and equity–an opportunity the healthcare sector can ill afford to miss.

Digital health technologies include using digital hardware and software in health care settings. The WHO in 2005 acknowledged the role of information and communication technology (ICT) in health and published a strategic plan 2020–2025 to ensure eHealth developed and was implemented in a way that promotes equity, affordability and access [2]. In this sense, innovation in eHealth includes new ideas, novel approaches and adaptations of existing ideas into the healthcare setting. Digital developments are the processes and continual improvements practically seen in ICTs. Therefore, eHealth has benefited from the development and continuous improvement in digital tools such as mobile phones and the internet, as well as the innovative application of technology to health [1,3]. Equally, this innovation has also been driven by rising healthcare costs, competing interests amongst stakeholders, a lack of cooperation across medical entities and global inequalities [4–6].

Importantly, the WHO strategic plan singles out equity as an imperative of eHealth. This important distinction highlights the need to take a social justice approach to digital health developments–when in design and planning stages, equity should be a foremost consideration (specifically not equality). Having said this, the current levels of inequality and inequality make it difficult to measure the impact of digital health, narrow down its significance, and create scalable solutions in the environments that need them the most. With the arrival of Covid-19, a substantial change in perspective regarding the adoption and implementation of digital health solutions has arisen. The pandemic emerged at a time when technology could augment or replace traditional healthcare interventions, and even created the opportunity for healthcare advances in the form of big data analysis and data sharing tools. These developments have created a broader platform for digital health interventions such as telehealth, online support, and supply chain system improvements to reach people unable to access health services, provided opportunities for advances in medical research, and created efficiencies in supply chain [7]. The question remains, however, have these advances in digital health effectively contributed to bridging the health inequity divide; or have the developments merely illustrated and perpetuated the health divide further?[7]

As with all systemic issues, the answer is both. The opportunity exists for us to now carefully consider the digital interventions that are available and to develop new interventions with the specific purpose of addressing health inequities; to implement solutions in a careful, considered, needs based manner; and to consider the economic, social, and developmental factors that will lead to the sustainability of the interventions.

## 1. Digital health is diverse–And its impact is unevenly dispersed

The Covid-19 lockdown forced people, and many components of healthcare systems, to move online, likely changing the face of healthcare in developed countries. The disruption was so pervasive and extreme, it created extensive buy-in and acceptance of the change. The support and adoption of the changes by public and private healthcare stakeholders cemented this acceptance [5]. However, the impact of using digital innovation to manage the pandemic was not equally experienced in developing countries with less developed infrastructure, lack of resources, fewer technical skills, inconsistent energy supply, and political focus on areas other than digital development. In both under-served areas in the developed world (see for instance WHO analysis of European region[8]) and in developing countries the actual use of technology in health developments has excluded some communities and populations from their benefits. This exclusion is not specifically due to the technology per se, but rather the requisite skills, human resources, infrastructure, policy, finance and enabling environment at large that needs to be in place for these technologies to be implemented.

There are many instances where digital health innovations have been implemented without due consideration of the social and healthcare systems into which they are being placed [1]. These areas must be addressed to bridge the digital divide or developing countries will risk missing the opportunities that digital health presents [9].

## 2. Digital health innovations can make a difference to health inequity, IF fit for purpose

Digital healthcare, intended to be inclusive, may entrench inequity by marginalizing those affected by poverty, poor adoption of digital healthcare innovations, lack of access to systems, and poor digital literacy [10]. In Uganda, a cross-sectional investigation of how patient records are stored found that an integrated system of patient information was hampered by diverse sources of data collection, inequal access to electronic systems, inadequate healthcare data systems and ICT, poor security and privacy safeguards and weak governance structures [11]. Sub-Saharan Africa, for instance, is severely under-resourced in healthcare professionals, equipment and resources, yet accounts for 24% of the global disease burden. This situation is worse in rural areas. Yet, there is no lack of examples of digital health solutions in Sub-Saharan Africa [12].

Health intervention research requires an equity evaluation which currently may not explicitly take place [13]. There are many instances where digital health innovations have been implemented without due consideration of the social and healthcare systems into which they are being placed. This results in small scale evaluations, setting-specific data, or lack of measurement of the impact of the intervention [1]. A review of 29 articles on costs, benefits, and economic indicators of eHealth interventions in low and middle-income countries found that measurement and impact information is scarce, small in scale and mostly focused on pilot projects. Overall feasibility and cost-effectiveness were not assessed by the studies [14]. For an enabling policy and strategy environment, this level of evidence is essential, and the lack thereof hampers adoption. In resource scarce environments, and with the emergence of various eHealth opportunities, the cost-benefit impact of interventions is essential for health systems to properly make decisions for implementation [1,14].

Covid-19 resulted in some very widespread examples of digital health innovation, from the emergence of tracking apps using artificial intelligence, to the use of big data to predict physical and mental health outcomes, to telehealth as lockdowns reduced access to healthcare professionals [7,15,16]. As a specific example, in the United States, telehealth was further progressed due to a relaxation or adaption of policy and legislation to allow its implementation, with associated reimbursement mechanisms [17]. The pandemic was a naturally occurring, large-scale proof-point for the efficacy and potential for eHealth solutions. Lockdown forced people, and many components of healthcare systems, to move online, likely changing the face of healthcare forever in developed countries. The disruption was so pervasive and extreme, it created extensive buy-in and acceptance of the change. The support and adoption of the changes by public and private healthcare stakeholders cemented this acceptance. This demonstrates two of the primary levers of success in implementing digital health innovations–stakeholder buy-in for the change, and adoption of the new technology by credible stakeholders [5]. Meta-analyses of the impact Covid-19 had, and continues to have, on the digital health landscape must be undertaken to determine the ‘stickiness’ of certain interventions, the factors that create this ‘stickiness’ and their impact. Imperative in these evaluations will be social context, cultural factors that are barriers to or opportunities for digital health interventions, and the policy and legislative environment. Future research should be undertaken within socio-cultural frameworks of adoption such as those posited by Crawford and Serhal[16] or similar protocols such as those used by Hadjiat and Perrot [18].

## 3. Design health interventions within an equity framework

Crawford and Serhal have developed a Digital Health Equity Framework which articulates several factors that should be considered when promoting digital health equity. These factors should be considered holistically as they are interrelated and can improve or exacerbate one another. They include:

- **Socio-economic and cultural contexts**—social stratification, social location (access to resources, prestige, and discrimination) and material circumstances.
- **Intermediate factors shaped by social context**—psychosocial stressors, appraisal and coping, biology (stress response, pre-existing health state), health-related beliefs and behaviors, health state and need, and environment.
- **Digital determination of health**–access to and use of digital resources, use of digital health literacy, beliefs about digital health, values, and cultural norms for using digital resources, and integration of digital resources into the community and health infrastructure.
- **Health system as a social determinant of health**—health policy and funding, governance, institutional policies and leadership, health education and training, and the patient-provider relationship.
- **Resourcing and quality of care**—access to care, timeliness of care, effectiveness, efficiency and safety of care, person-centered care, community-centered care, and cultural safety of care; and
- **Digital health equity**—equal access to digital healthcare and equal outcomes from digital healthcare irrespective of age, gender, ethnicity, income and geography, health providers with competencies/training to provide digital healthcare, measurement and quality improvement, and involvement of people from vulnerable groups [16].

This framework emphasizes the point that digital health research and innovation must consider social factors and health equity. This consideration must start with policy and regulation for digital health that has the objective of addressing health inequity, with clear steps to address and consider factors of need, relevance, and inclusion [16]. In addition, patients and marginalized communities must be included in developing solutions to meet their needs [6,19,20]. Importantly, the framework is clear that merely accessing healthcare is not a determinant of good health outcomes. Social facilities such as food security, transportation and community support are all contributing factors in health outcomes [21].

To be truly inclusive, digital healthcare must go beyond digitizing what exists and must pursue digitalization at scale [22]. It is essential that adoption of digital healthcare occurs hand-in-hand with community driven need for the solutions and digital literacy education [5]. Digital healthcare will face barriers from fundamental infrastructure challenges such as internet access, interoperability, costs, access to technology, and even access to electricity [5]. The potential of digital healthcare to positively impact the lives of people in both developed and developing countries is a call to action for all working in this field of study. Contextual relevance and deliberate action to bridge inequities is called for if further erosion of the chasm between the ‘haves’ and the ‘have-nots’ is to be avoided.
